# Differing associations of BMI and body fat with asthma and lung function in children

**DOI:** 10.1002/ppul.22927

**Published:** 2013-10-25

**Authors:** Ran Wang, Adnan Custovic, Angela Simpson, Danielle C Belgrave, Lesley A Lowe, Clare S Murray

**Affiliations:** 1 Manchester Academic Health Science Centre, University of Manchester, University Hospital of South Manchester, NHS Foundation TrustManchester, UK

**Keywords:** body mass index, bioelectrical impedance, pulmonary function, asthma, adiposity

## Abstract

**Summary:**

**Background:**

Current evidence suggests that in children there is a significant, albeit weak, association between asthma and obesity. Studies generally use body mass index (BMI) in evaluating body adiposity, but there are limitations to its use.

**Method:**

Children from a population-based study attending follow-up (age 11 years) were weighed, measured and had percent body (PBF) and truncal (PTF) fat assessed using bioelectrical impedance. They were skin prick tested and completed spirometry. Parents completed a validated respiratory questionnaire. Children were defined as normal or overweight according to BMI and PBF cut-offs. We tested the association between these adiposity markers with wheeze, asthma, atopy, and lung-function.

**Results:**

Six hundred forty-six children (339 male) completed follow-up. BMI *z*-score, PBF, and PTF were all positively associated with current wheeze (odds ratio [95% CI]: 1.27 [1.03, 1.57], *P* = 0.03; 1.05 [1.00, 1.09], *P* = 0.03; 1.04 [1.00, 1.08], *P* = 0.04, respectively). Similar trends were seen with asthma. However, when examining girls and boys separately, significant positive associations were found with PBF and PTF and asthma but only in girls (gender interaction *P* = 0.06 and 0.04, respectively). Associations between being overweight and wheezing and asthma were stronger when overweight was defined by PBF (*P* = 0.007, 0.03) than BMI (*P* > 0.05). Higher BMI was significantly associated with an increase in FEV_1_ and FVC, but only in girls. Conversely, increasing body fat (PBF and PTF) was associated with reduced FEV_1_ and FVC, but only in boys. No associations between adiposity and atopy were found.

**Conclusion:**

All adiposity measures were associated with wheeze, asthma, and lung function. However, BMI and PBF did not have the same effects and girls and boys appear to be affected differently. **Pediatr Pulmonol. 2014; 49:1049–1057.**

## INTRODUCTION

The UK prevalence of asthma and allergic disease in children ranks among the highest in the world.^1^ With a simultaneous epidemic of childhood obesity,^2^ associations between obesity and asthma and atopy have been suggested. Recent systematic reviews and data from large epidemiological studies suggest a significant association between asthma and obesity^3^–^5^ but the evidence for an association between atopy and obesity remains conflicting.^6^–^8^ While lung function deficits have been observed in overweight and obese adults,^9^,^10^ these findings have not been universally reported in children.^11^–^14^

As most of the studies investigating the association between asthma/atopy and obesity have used Body mass index (BMI) as a fat indicator, the results of studies may reflect the limitation of BMI to predict body adiposity and health risks in children; BMI can neither distinguish muscle mass from fat mass, nor can it measure fat distribution. With a given BMI, girls also have significantly higher amounts of percentage body fat than boys.^15^ Although two studies have used skin fold thickness as direct measure of body fat,^16^,^17^ they have shown inconsistent results.

Bioelectrical impedance analysis (BIA) is a relatively new method to examine body composition. It determines the electrical impedance, or opposition to the flow of a small electrical current through body tissues which can then be used to calculate an estimate of total body water. This is then used to estimate fat-free body mass, and by difference with body weight, body fat can be derived.^18^ It is cheap, practical, and non-invasive. Although BIA has been used in studies investigating the association between adiposity and asthma in adults^19^ it has not yet been used in such studies in children.

We hypothesize that, within a normal population, different measures of body adiposity (BMI and body fat assessed by BIA) will show different associations with wheeze, asthma, and lung function.

## MATERIALS AND METHODS

### Study Design, Setting, and Participants

The Manchester Asthma and Allergy Study is a population-based birth cohort study described in detail elsewhere.^20^ Briefly, parents were consented and screened for eligibility at antenatal clinics. Children were followed from birth, and attended review clinics at age 1, 3, 5, 8, and 11–12 years. The study protocol was approved by the Local Research Ethic Committee, and all parents gave written informed consent. In this manuscript, we report on cross-sectional data collected at age 11–12 years.

### Data Sources

#### Clinical follow-up

Participants attended review clinics at age 11–12 years (mean age 11.5 years, SD 0.5). Parents completed an interviewer-administered validated questionnaire.^21^ Atopic sensitization was ascertained by skin-prick tests (*Dermatophagoides pteronyssinus*, cat, dog, grasses, moulds, milk, and egg; Bayer, Elkahrt, IN). Lung function was assessed by spirometry and was measured before (pre-bronchodilator) and 15 min after administration of 400 μg of salbutamol (post-bronchodilator).^22^

#### Growth and adiposity measurements

We measured weight, without shoes or outer clothing (to nearest 0.01 kg), using a weighing scale, and height (to nearest 0.1 cm) using a stadiometer. Percent body fat (PBF) and percent truncal fat (PTF) were measured by the hand-to-foot 8-electrode bioelectrical impedance analyzer (Tanita BC-418, Tokyo, Japan [BIA_8_]).

Physical development was assessed using diagrams of Tanner stages^23^; these were developed at the University of North Carolina's Population Center and have been previously validated and successfully used in other cohort studies.^24^ Participants were then classified as pre-pubescent (mean score ≤1) or peri-pubescent (mean score >1) according to mean score for pubic hair and breast/testes development.

### Definition of Variables

#### Current wheeze

Defined as a positive response to the question “Has your child had wheezing or whistling in the chest in the last 12 months?”

#### Current asthma

Defined as history of physician-diagnosed asthma and wheezing in the last 12 months.

#### Atopy

Children were skin-prick tested and were classified as atopic if the weal diameter to any allergen was 3 mm or more greater than the negative control.

#### Markers of adiposity

BMI was calculated as weight/height^2^ (kg/m^2^). We present BMI as standardized deviation scores (BMI *z*-score) using the British Growth Reference (1990).^25^ We designated each individual as “normal weight” or “overweight” using previously published BMI^26^ and PBF^27^ age and gender specific cut-off values.

#### Lung function

Forced expiratory volume in one second (FEV_1_) and forced vital capacity (FVC) were recorded.

### Statistical Analysis

Analysis was carried out using SPSS 19 (Chicago, IL). We used logistic regression to model binary outcomes (current wheeze, asthma, and atopy). Linear regression was used to determine the effects of increased body adiposity on lung function. Confounders with *P*-value ≤0.1 in univariate analysis were included in the multivariate analysis.

Within this cohort study, within each gender group, we have estimated that we have 95% power to detect a difference in BMI *z*-score of 0.5 in wheezy boys/girls compared with non-wheezy boys/girls, at the 5% significance level. Similarly for PBF and PTF we have over 80% power to detect a 2% difference between the groups at the 5% level of significance.

#### Missing data

Missing data were assumed to be missing completely at random. Under this assumption, analysis was carried out only on children with complete data.

#### Potential confounders

Ethnicity, sex, pre/peri-pubertal status, breastfeeding, birth weight, gestational age, parental asthma and atopy, environmental tobacco smoke, pets, child's atopy, and socio-economic status (National Statistics-Socio-economic Classification; http://www.ons.gov.uk/ons/guide-method/classifications/current-standard-classifications/soc2010/soc2010-volume-3-ns-sec-rebased-on-soc2010-user-manual/index.html) were considered as confounders.

There were no subjects with endocrine disorders or long-term oral steroid use in the included population.

## RESULTS

### Study Population

**Fig 1:**
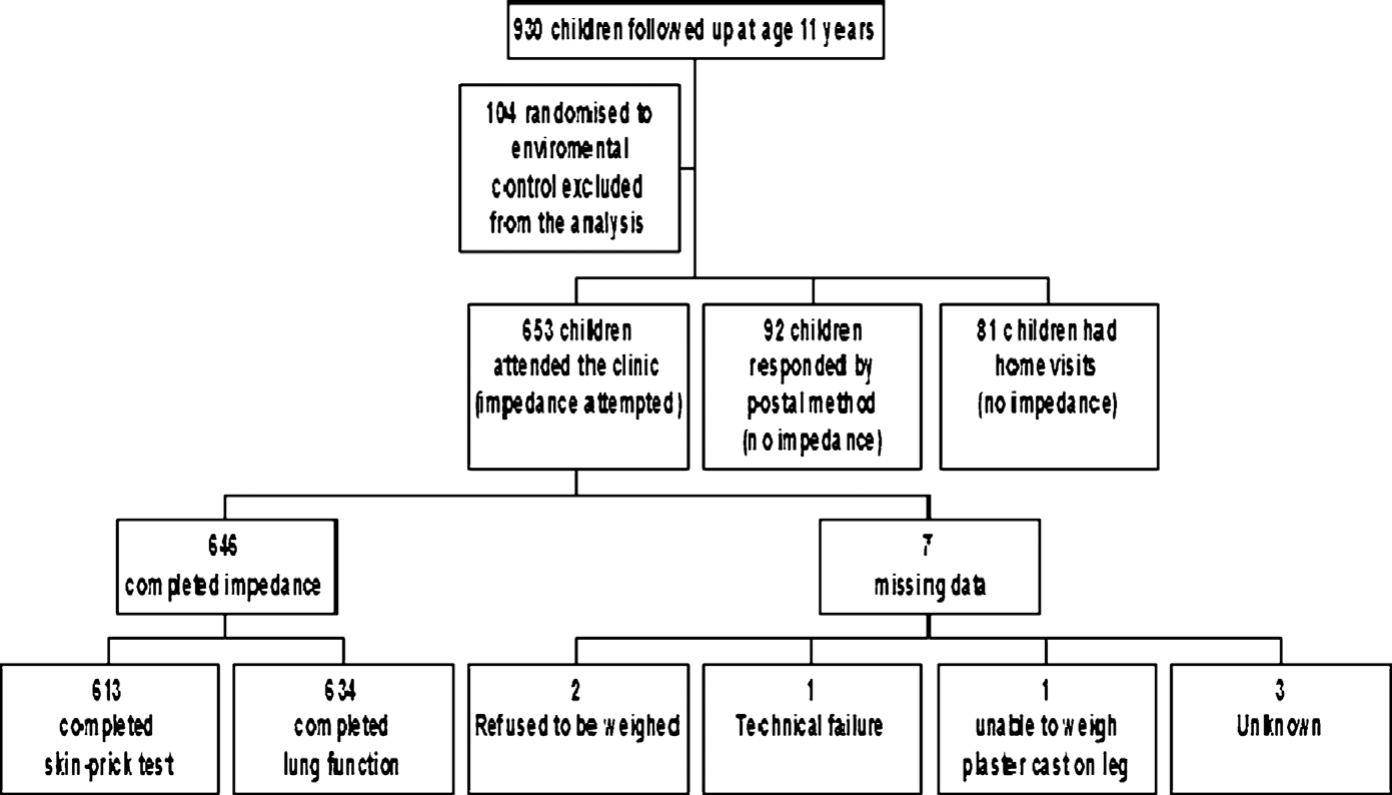
Consort diagram of data collected at age 11 follow-up visit.

Data were collected on 930 individuals at the age 11-year review; 104 children prenatally randomized to an environmental intervention^20^ were excluded from this analysis. Of 826 children in the observational cohort, 653 attended the hospital review and had body fat assessed using BIA (92 completed postal questionnaires, 81 home visits); 646 successfully completed BIA measurement (Fig. 1). There was no significant difference in asthma, BMI, ethnicity, socio-economic status, birth weight, gestational age, and parental atopy between children included and not included in the analysis. Demographics of the study population are shown in Table1. There were clear differences between boys and girls in the prevalence of asthma and atopy and also in measures of body fat; subsequent analysis was therefore also conducted in boys and girls separately.

### Association Between Measures of Adiposity and Clinical Outcomes

#### Current wheeze

In the univariate analysis, higher BMI was associated with an increased risk of wheezing (OR [95% CI]: 1.21 [1.01–1.44], *P* = 0.04); there was no association between wheeze and PBF or PTF (Table2). However, when girls and boys were examined separately, we found that wheeze in girls was significantly associated with higher PBF and PTF. In the multivariate analysis, BMI, PBF and PTF were all significantly associated with wheeze (Table3). Although the association between adiposity and wheeze appeared stronger in girls, there was no significant interaction with gender.

When children were classified as overweight or normal weight according to PBF, the risk of wheeze significantly increased with being overweight (1.99 [1.32–3.01], *P* = 0.001; E-Table S1). This remained significant in the multivariate analysis (*P* = 0.007; Table4). When boys and girls were examined separately, overweight girls were at significantly increased risk of wheeze (2.65 [1.13–6.23], *P* = 0.03), with no such association found in boys (1.47 [0.83–2.63], *P* = 0.19). Again, although inspection of data suggested that the association between markers of adiposity and wheeze was present in girls, but not boys, there was no significant interaction with gender (Table4).

#### Current asthma

When stratified by sex, all measures of body adiposity (BMI, PBF, and PTF) were significantly higher in girls with asthma; no such association was found in boys (Table2). In the multivariate analysis, there was a significant interaction between gender and PTF on the risk of current asthma (Table3), in that the risk of asthma significantly increased with increasing PTF in girls (1.10 [1.02–1.18], *P* = 0.01), with no such association in boys (1.00 [0.95–1.06], *P* = 0.91).

When children were categorized as overweight or normal weight according to PBF cut-offs, current asthma was significantly associated with being overweight (1.73 [1.10–2.83], *P* = 0.02; E-Table S1). After adjustment, this association remained significant (*P* = 0.03; Table4). This association was markedly stronger amongst girls (*P* = 0.08 for interaction), with odds for overweight girls having asthma being more than 3-fold higher (3.37 [1.34–8.48]) higher, and no increase in the risk of asthma among overweight boys (1.21 [0.61–2.40], Table4).

#### Atopy

We found no association between atopy and increased body adiposity (Tables4, E-Table S1).

#### Lung function

BMI was positively associated with FEV_1_ and FVC (Table5). These associations were similar both pre-bronchodilator (E-Table S2) and post-bronchodilator (Table5); the strongest relationships were noted for FVC. When stratified by gender the relationship between BMI and lung function was only present in girls where increasing BMI was associated with increased FEV_1_ and FVC. No significant associations were seen overall between PBF and PTF and lung function, either pre or post bronchodilator (E-Table S2 and Table5). When boys and girls were examined separately, no association was seen in girls. However, in boys increasing PBF and PTF resulted in a reduction of both FEV_1_ and FVC. Pre bronchodilator, this effect was not significant, but post-bronchodilator significant reductions were seen in both parameters such that a 5% increase in PTF was associated with 35 ml/sec reduction in FEV_1_ and 35 mL reduction in FVC (Table5).

**Table 1:** Baseline Characteristics of Children Who Attended the Age 11 Review With Impedance Measurements

	Overall (n = 646)	Boys (n = 339)	Girls (n = 307)	*P*-Value^*^
Age at follow up [years]	11.43 (0.53)	11.40 (0.55)	11.47 (0.50)	0.10
Anthropometric data				
Gestational age [weeks]	39.84 (1.64)	39.76 (1.75)	39.93 (1.51)	0.21
Birth weight [kg]	3.47 (0.51)	3.54 (0.51)	3.40 (0.49)	**<0.001**
Weight [kg]	42.43 (10.10)	41.62 (10.08)	43.32 (10.06)	0.33
Height [cm]	148.1 (7.66)	147.2 (7.78)	149.1 (7.41)	**0.002**
Body mass index [kg/m^2^]	19.16 (3.28)	19.01 (3.26)	19.33 (3.31)	0.22
Body mass index *z*-score	0.50 (1.11)	0.58 (1.11)	0.41 (1.10)	**0.044**
% Overweight according to BMI (n)	17.6 (114)	17.1 (58)	18.2 (56)	0.63
% Obese according to BMI (n)	5.4 (35)	6.2 (21)	4.6 (14)	0.63
Percent body fat	23.9 (6.19)	22.1 (6.39)	25.9 (5.30)	**<0.001**
% Overweight according to PBF (n)	26.9 (174)	36.3 (123)	16.6 (51)	**<0.001**
% Obese according to PBF (n)	2.9 (19)	4.7 (16)	1 (3)	**<0.001**
Percent truncal fat	18.5 (6.16)	17.5 (6.47)	19.6 (5.59)	**<0.001**
Asthma and atopy				
% Atopy sensitization (n)	32.3 (209)	38.1 (129)	26.1 (80)	**0.002**
% Current wheeze (n)	18.6 (120)	23 (78)	13.7 (42)	**<0.001**
% Current asthma (n)	12.8 (83)	15.9 (54)	9.4 (29)	**0.02**
Lung function	Overall (n = 635)	Boys (n = 333)	Girls (n = 302)	*P*-Value^*^
Baseline FEV_1_ [L/sec]	2.32 (0.41)	2.32 (0.41)	2.32 (0.40)	0.87
Baseline FVC [L]	2.69 (0.49)	2.74 (0.52)	2.62 (0.46)	**0.002**
	Overall (n = 625)	Boys (n = 325)	Girls (n = 300)	*P*-Value^*^
Post-BD FEV_1_ [L/sec]	2.45 (0.41)	2.47 (0.40)	2.44 (0.41)	0.42
Post-BD FVC [L]	2.73 (0.49)	2.79 (0.49)	2.67 (0.48)	**0.003**
Pubertal stage	Overall (n = 544)	Boys (n = 269)	Girls (n = 275)	*P*-Value^*^
% Peri-pubertal (n)	80 (436)	80 (216)	80 (220)	0.93
Mean Tanner's stage	2.04 (0.867)	1.96 (0.795)	2.12 (0.926)	**0.03**

Data presented as means (standard deviation) for continuous variables or percentage (number) for categorical variables; *P*-value^*^ comparison between genders, based on *t*-test for continuous variables and *χ*^2^-test for categorical variables; bold signifies *P* < 0.05.

Post-BD, post-bronchodilator.

When examining children with current wheeze, history of early childhood wheeze, self-reported asthma, and healthy children separately, similar patterns were observed (data not shown).

## DISCUSSION

### Key Findings

This is the first study to report significant associations between increasing body adiposity in children, as measured by bioelectrical impedance, and asthma, wheeze, and lung function.

There were marked differences in the prevalence of asthma and atopy by gender at age 11 years, with both being significantly more common in boys. Also although girls had a higher percentage body fat than boys, they were less likely to be considered overweight by this parameter, reflecting the differences in normal ranges by gender.

Importantly, we found no association between adiposity and atopy. Although for boys there was no association between measures of adiposity and wheeze or asthma, for girls adiposity strongly and consistently predicted asthma and wheeze. The effect size for being overweight by PBF definition was greater than that seen for being overweight by BMI criteria. Girls who were overweight by PBF-defined criteria were more than three times as likely to have current asthma and 2.5 times more likely to wheeze than normal-weight girls.

Furthermore, for each 1% increase in body fat, there was a 10% increase in risk of asthma. However, it is important to note that these findings are based primarily on analysis of boys and girls separately and a statistically significant gender interaction was only found for asthma and PTF.

For lung function, BMI was significantly associated with an increase (albeit small) in FEV_1_ and FVC only in girls. Conversely, increasing body fat (PBF and PTF) was associated with reduced FEV_1_ and FVC, but only in boys. These effects were evident both before and after the administration of 400 μg of salbutamol, but were only statistically significant after bronchodilation. It is noteworthy that the associations of adiposity were stronger with post- rather than pre-bronchodilator lung function; we believe this supports the view that effects of adiposity on lung function are independent of asthma or wheeze.

**Table 2:** BMI, PBF, and PTF in Relation to Current Wheeze, Asthma, and Atopy

	BMI *z*-Score	*P*-Value	PBF	*P*-Value	PTF	*P*-Value
Overall						
Wheeze, n = 120	0.69 (1.17)	**0.04**	24.38 (6.54)	0.37	19.09 (6.45)	0.21
No wheeze, n = 526	0.46 (1.09)		23.82 (6.11)		18.31 (6.09)	
Current asthma, n = 83	0.70 (1.15)	0.07	24.28 (6.99)	0.58	19.01 (6.83)	0.39
No current asthma, n = 563	0.47 (1.10)		23.88 (6.07)		18.38 (6.05)	
Atopic, n = 209	0.52 (1.18)	0.83	23.90 (6.43)	0.74	18.63 (6.23)	0.78
Non-atopic, n = 404	0.50 (1.08)		24.07 (6.14)		18.48 (6.19)	
Boys						
Wheeze, n = 78	0.69 (1.10)	0.35	22.61 (6.23)	0.43	17.77 (6.29)	0.62
No wheeze, n = 261	0.55 (1.12)		21.97 (6.44)		17.36 (6.53)	
Current asthma, n = 54	0.66 (1.06)	0.61	22.06 (6.40)	0.95	17.23 (6.47)	0.79
No current asthma, n = 285	0.57 (1.13)		22.13 (6.40)		17.49 (6.48)	
Atopic, n = 129	0.57 (1.15)	0.61	22.35 (6.38)	0.79	17.69 (6.21)	0.77
Non-atopic, n = 190	0.63 (1.10)		22.15 (6.56)		17.47 (6.79)	
Girls						
Wheeze, n = 42	0.69 (1.31)	0.07	27.7 (5.84)	**0.02**	21.55 (6.07)	**0.01**
No wheeze, n = 265	0.36 (1.06)		25.66 (5.16)		19.26 (5.46)	
Current asthma, n = 29	0.79 (1.32)	**0.05**	28.40 (6.19)	**0.008**	22.31 (6.33)	**0.005**
No current asthma, n = 278	0.37 (1.07)		25.67 (5.14)		19.29 (5.45)	
Atopic, n = 80	0.45 (1.23)	0.66	26.39 (5.71)	0.38	20.15 (6.00)	0.30
Non-atopic, n = 214	0.39 (1.05)		25.78 (5.18)		19.38 (5.47)	

Data are presented as mean (standard deviation); *P* values were calculated using *t*-test; bold signifies *P* < 0.05.

### Interpretation of Findings

Many previous studies from around the world have reported that increasing BMI is associated with wheeze and asthma and that the effect is stronger in girls than boys.^6^,^12^,^16^,^28^,^29^ One study using BIA to assess body fat in adults reported a significant association between higher PBF and asthma, but only in women.^19^ Two pediatric asthma studies have used skin fold thickness as an alternative measure of fatness.^16^,^17^ Figueroa-Munoz et al.^16^ reported no association between the sum of triceps and sub-scapular skin fold thicknesses and asthma. Cassol et al.^17^ used triceps skin fold thickness in combination with BMI to define obesity and found a significant positive association with wheeze ever, asthma ever and severe asthma. To our knowledge, ours is the first study to assess PBF and PTF using BIA in children and investigate its association with asthma and wheeze. Although both higher BMI and PBF predicted asthma in girls the association between body fat and asthma/wheeze was stronger than the association with BMI. It is important to note that BMI cannot distinguish between and fat and muscle mass and we note that in girls and boys the relation between body fat and BMI is different. Girls have higher percentage of body fat for each unit of BMI (E-Fig. S1). Therefore the association appears to be between fat (rather than weight) and asthma in girls; we would therefore advocate the use of fat measurement as opposed to BMI when investigating these associations.

**Table 3:** Multivariate Analysis of the Associations Between Wheeze, Asthma, and Atopy and Body Adiposity (BMI *z*-Score, PBF, and PTF)

	BMI *z*-Score		PBF		PTF	
	OR [95% CI]	*P*-Value (*P*_Int_)	OR [95% CI]	*P*-Value (*P*_Int_)	OR [95% CI]	*P*-Value (*P*_Int_)
Overall						
Wheeze^1^ (n = 646)	1.27 [1.03–1.57]	**0.03** (0.46)	1.05 [1.00–1.09]	0.03 (0.19)	1.04 [1.00–1.08]	**0.04** (0.11)
Current asthma^1^ (n = 646)	1.25 [0.99–1.57]	0.06 (0.38)	1.03 [0.99–1.07]	0.16 (0.06)	1.03 [0.99–1.08]	0.12 (**0.04**)
Atopy^2^ (n = 613)	1.02 [0.97–1.08]	0.39 (0.97)	1.02 [0.99–1.05]	0.21 (0.95)	1.02 [0.99–1.05]	0.19 (0.99)
Boys						
Wheeze^2^ (n = 339)	1.17 [0.91–1.51]	0.22	1.02 [0.98–1.07]	0.33	1.02 [0.97–1.06]	0.51
Current asthma^2^ (n = 339)	1.15 [0.86–1.55]	0.35	1.01 [0.96–1.06]	0.75	1.00 [0.95–1.06]	0.91
Atopy^2^ (n = 319)	1.04 [0.84–1.28]	0.74	1.02 [0.98–1.06]	0.34	1.02 [0.98–1.06]	0.33
Girls						
Wheeze^1^ (n = 307)	1.40 [0.97, 2.01]	0.07	1.07 [0.99, 1.14]	0.07	1.07 [1.00, 1.14]	**0.05**
Current asthma^1^ (n = 307)	1.40 [0.93, 2.01]	0.08	1.10 [1.02, 1.18]	**0.02**	1.10 [1.02, 1.82]	**0.01**
Atopy^2^(n = 294)	1.03 [0.81, 1.31]	0.82	1.01 [0.97, 1.07]	0.57	1.02 [0.97, 1.07]	0.48

Bold signifies *P* < 0.05. OR, odds ratio; 95% CI, 95% confidence interval; *P*_Int_: *P*-value for interaction between BMI *z*-score/PBF/PTF and gender.

1 Table 1Adjusted for parental asthma, child's atopy and gender (where applicable).

2 Table 2Adjusted for presence of dog at home, maternal and paternal atopy and gender (where applicable).

**Table 4:** Odds Ratios for Asthma, Wheeze, and Atopy in Overweight Children-Multivariate Logistic Regression: Using Normal Weight as the Reference Category

	BMI-defined			PBF-defined		
	OR	[95% CI]	*P*-Value (*P*_Int_)	OR	[95% CI]	*P*-Value (*P*_Int_)
Overall						
Wheeze^1^ (n = 646)	1.51	0.89–2.56	0.13 (0.52)	1.93	1.19–3.11	0.007 (0.26)
Current asthma^1^ (n = 646)	1.65	0.93, 2.94	0.09 (0.21)	1.84	1.07, 3.16	**0.03** (0.08)
Atopy^2^ (n = 613)	1.22	0.81–1.84	0.34 (1.00)	1.26	0.85–1.85	0.24 (0.36)
Boys						
Wheeze^1^ (n = 339)	1.35	0.70–2.60	0.37	1.47	0.83–2.63	0.19
Current asthma^1^ (n = 339)	1.22	0.57–2.64	0.61	1.21	0.61–2.40	0.58
Atopy^2^ (n = 319)	1.21	0.70–2.10	0.49	1.10	0.68–1.78	0.69
Girls						
Wheeze^1^ (n = 307)	1.93	0.81–4.64	0.14	2.65	1.13–6.23	**0.03**
Current asthma^1^ (n = 307)	2.56	1.03–6.08	**0.04**	3.37	1.34–8.48	**0.01**
Atopy^2^ (n = 294)	1.21	0.65–2.25	0.55	1.65	0.86–3.16	0.13

Bold signifies *P* < 0.05. OR, odds ratio; 95% CI, 95% confidence interval; *P*_Int_: *P*-value for interaction between BMI *z*-score/PBF/PTF and gender.

1 Table 1Adjusted for parental asthma, child's atopy and gender (where applicable).

2 Table 2Adjusted for maternal and paternal atopy, dog presence at home and gender (where applicable).

Previous studies investigating the relationship between lung function and obesity in children have reported conflicting results. A population based study of Chinese school-children aged 6–20 years using BMI as a marker of adiposity found similar results to our study, reporting a positive correlation between BMI and lung function.^14^ A US study of mild to moderate asthmatic children aged 5–12 years also found that increasing BMI was associated with increases in FEV_1_ and FVC.^11^ Consistent with our findings, both these studies reported stronger associations with BMI and lung function in girls.

**Table 5:** Associations of Body Adiposity (BMI, PBF, and PTF) With Post-Bronchodilator Lung Function—Multiple Linear Regression

	Overall (n = 544)			Boys (n = 265)			Girls (n = 279)		
	β	95% CI	*P*-Value	β	95% CI	*P*-Value	β	95% CI	*P*-Value
BMI *z*-score^1^									
FEV_1_ (L)	0.034	0.013, 0.055	**0.002**	0.017	−0.015, 0.048	0.30	0.036	0.008, 0.065	**0.01**
FVC (L)	0.059	0.031, 0.087	**<0.001**	0.021	−0.022, 0.063	0.34	0.068	0.034, 0.102	**<0.001**
BMI^2^									
FEV_1_ (L)	0.009	0.002, 0.017	**0.009**	0.005	−0.005, 0.015	0.31	0.010	0.001, 0.019	**0.03**
FVC (L)	0.017	0.007, 0.026	**<0.001**	0.008	−0.006, 0.021	0.26	0.020	0.010, 0.031	**<0.001**
PBF^2^									
FEV_1_ (L)	−0.003	−0.007, 0.001	0.14	−0.006	−0.011, −0.001	**0.02**	−0.002	−0.007, 0.004	0.52
FVC (L)	−0.001	−0.006 0.004	0.77	−0.006	−0.013, 0.000	0.06	0.003	−0.003, 0.009	0.33
PTF^2^									
FEV_1_ (L)	−0.004	−0.007, 0.000	**0.05**	−0.007	−0.012, −0.002	**0.004**	−0.002	−0.007, 0.003	0.38
FVC (L)	−0.002	−0.006, 0.003	0.47	−0.007	−0.014, −0.001	**0.02**	0.002	−0.004, 0.008	0.44

Bold signifies *P* < 0.05. β, Estimated effect size; 95% CI, 95% confidence intervals.

1 Table 1Adjusted for pre/peri-pubertal status and height.

2 Table 2Adjusted for pre/peri-pubertal status, exact age, and height.

Overall Regression also adjusted for gender where appropriate.

However, in a study of mainly obese children (aged 6–11 years) FEV_1_ and FVC were significantly reduced in overweight/obese children compared to children with normal weight.^12^ Another small study of obese children found no correlation between BMI and functional residual capacity (FRC).^13^ Neither study examined boys and girls separately.

The inconsistent findings in the association between lung function in children and BMI^11^–^13^ seems to indicate that findings are dependent on which population is studied. If one studies a population in which BMI is normally distributed, increasing BMI predicts better lung function. However, when an obese population of children is studied very high BMI is associated with poorer lung function, indicating that the relationship between BMI and lung function may not linear. In our population, only 35 children were classed as obese (approx. 5%) and of these only 4 had BMI above 30 kg/m^2^ (the usual obese cut-off for adults); the heaviest had a BMI of 32.9 kg/m^2^. Thus it is unlikely that our observations would be the same as those studies of obese individuals^13^ or to those with high proportion of overweight/obese children.^12^

In contrast to our findings for BMI, we found that for boys but not girls, increasing PBF and PTF predicted poorer lung function, where no association was seen for BMI. It is of note that obese boys deposit fat in the abdomen, whilst girls deposit fat in the sub-scapular area.^30^ Abdominal fat may decrease the expiratory reserve volume, thus reducing the FVC and FEV_1_. We note that for boys in our study the effects on lung function were greatest for truncal fat.

This study demonstrates that there are different directions of association for BMI and PBF/PTF and lung function. It would appear that BMI reflects body size/growth, rather than fat mass, except perhaps in very obese children, whereas assessing body fat by bioelectrical impedance may be a better and more specific measure of fatness itself.

Thus, we would advocate measuring both BMI and body fat and analyzing genders separately in future studies of this type.

It is plausible that adiposity effects lung physiology and disease by different mechanisms. For example, truncal distribution of adipose tissue could have a mechanical effect on respiratory physiology as measured by lung function. It is also well recognized that adipokines for example leptin acts on inflammatory cells including eosinophils.^31^ The gender differences that we have demonstrated for both symptoms and disease (wheeze and asthma) and lung function may be the result of both the amount and/or distribution of adipose tissue. When approaching puberty, girls tend to have higher body fat composition than boys for an equivalent BMI.^15^ This may mean that the association between increased body adiposity and asthma and wheeze can be more readily observed in girls.

It has also been suggested that the increases in estrogen and progesterone levels during menarche may also play a role in the gender differences seen.^32^ In our study mean Tanner stage was significantly higher in girls than boys.

### Strengths and Limitations

The present study used different body adiposity measures (BMI *z*-score, PBF, and PTF) to investigate the association with wheeze, asthma and lung function. We used BIA to measure PBF and PTF. The 8-electrode BIA_8_ device has been shown to have acceptable accuracy and be a reliable field assessment technique in studies comparing it with DEXA.^33^–^35^ Compared to the previously used skin-fold thickness method, BIA has minimal inter-operator variability and causes less discomfort to the subjects. The raw data are reproducible, with <1% error on repeated studies.^36^ A recent study compared anthropometric measurements to BIA and showed good correlation between the two^37^ however, BIA is quicker and easier to perform.

The use of PBF and PTF in studies of overweight children may have advantages over just using BMI. They better indicate fat mass and fat distribution as opposed to total mass (fat mass and muscle mass). Thus PBF and PTF can more accurately demonstrate the associations of body adiposity with risk of outcome. For example, the increased FEV_1_ and FVC observed with increased BMI may well be due to increased muscle mass in those with higher BMI, or a reflection of increased body growth resulting in increased lung volumes. When PBF and PTF were used as markers of adiposity, reduced FEV_1_ and FVC were observed.

This study is a cross-sectional study and therefore it is only able to suggest association rather than causality between adiposity measures and asthma and lung function.

International pediatric age/gender specific BMI cut-offs are well established for defining overweight and obesity,^26^ however, pediatric BIA-derived PBF cutoff values are not available. In order to define overweight/obese by PBF we used published DEXA-derived PBF cutoffs.^27^ Hence our weight classification may have been influenced by any BIA_8_–DEXA discrepancies.^35^

Although the total number of subjects included in the study is quite large we acknowledge that the number of cases is relatively small, particularly when boys and girls are assessed separately. However, power calculations show we are sufficiently powered to detect what we believe to be a clinically significant difference, thus reducing the type II error.

We adjusted our results for a number of potential confounders (Ethnicity, pre/peri-pubertal status, breastfeeding, birth weight, gestational age, parental asthma and atopy, environmental tobacco smoke, pets, child's atopy, and socio-economic status). However, we were unable to consider physical activity and dietary factors in our analysis.

Associations reported between wheeze and current asthma and BIA measurements appeared to differ between girls and boys. However, it is important to note that these findings are based primarily on analysis of boys and girls separately and a statistically significant gender interaction was only found for asthma and PTF, and thus these results should be interpreted with caution.

## CONCLUSION

We found significant associations between body adiposity (BMI, PBF, and PTF) and wheeze and asthma at age 11 years, which appeared to be stronger in girls and also stronger for percent fat than BMI measurements. BMI and PBF and PTF were significantly associated with FEV_1_ and FVC, but the observations were different for BMI and percent fat. We believe the mechanisms for the associations demonstrated between adiposity and wheeze/asthma and lung function are likely to be different (pro-inflammatory versus mechanical).

This study highlights the differences between BMI and body fat measurements and also the variation in body fat between the sexes. Thus for future studies of this type we would recommend measuring both percent fat and BMI and investigating gender differences.

## Supporting Information

Supplementary **Fig. S1**. Plot for body fat percentage and BMI values for children at age 11 years. Blue triangles, boys; red circles, girls. **TABLE S1.** Odds Ratios for Wheeze, Asthma, and Atopy in Overweight Children -Univariate Logistic Regression: Using Normal Weight as the Reference Category **TABLE S2.** Associations of Body Adiposity (BMI, PBF, and PTF) With Pre-Bronchodilator Lung Function—Multiple Linear Regression
