# Supplementary figures and images for: Differing associations of BMI and body fat with asthma and lung function in children
Source: Pediatr Pulmonol. 2013 Oct 25;49(11):1049–57. doi: 10.1002/ppul.22927 (PMC4265846; doi:10.1002/ppul.22927)

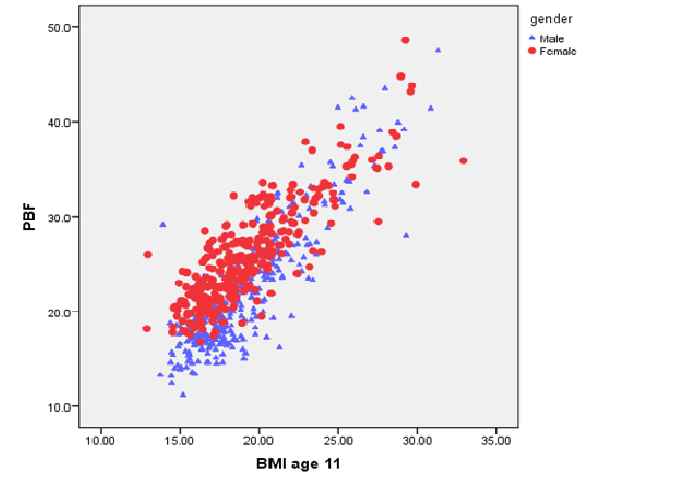

Supplement: Supplementary file 1 — Supplementary Fig. S1. Plot for body fat percentage and BMI values for children at age 11 years. Blue triangles, boys; red circles, girls. TABLE S1. Odds Ratios for Wheeze, Asthma, and Atopy in Overweight Children -Univariate Logistic Regression: Using Normal Weight as the Reference Category TABLE S2. Associations of Body Adiposity (BMI, PBF, and PTF) With Pre-Bronchodilator Lung Function—Multiple Linear Regression [file ppul0049-1049-sd1.tif]
