# Supplementary material for: Differing associations of BMI and body fat with asthma and lung function in children
Source: Pediatr Pulmonol. 2013 Oct 25;49(11):1049–57. doi: 10.1002/ppul.22927 (PMC4265846; doi:10.1002/ppul.22927)
Supplement: Supplementary file 2 — Supplementary Fig. S1. Plot for body fat percentage and BMI values for children at age 11 years. Blue triangles, boys; red circles, girls. TABLE S1. Odds Ratios for Wheeze, Asthma, and Atopy in Overweight Children -Univariate Logistic Regression: Using Normal Weight as the Reference Category TABLE S2. Associations of Body Adiposity (BMI, PBF, and PTF) With Pre-Bronchodilator Lung Function—Multiple Linear Regression [file ppul0049-1049-sd2.docx]

**E-Table 1.** *Odds ratios for wheeze, asthma and atopy in overweight children -univariate logistic regression: using normal weight as the reference category*

| Overall | **BMI-defined** | | | **PBF-defined** | | | | |
| --- | --- | --- | --- | --- | --- | --- | --- | --- |
|  | **OR** | **[95%CI]** | ***p*-value^α^** | | **OR** | | **[95%CI]** | ***p*-value^α^** |
| **Wheeze**  **(N=646)** | 1.41 | 0.90-2.21 | 0.13 | | 1.99 | | 1.32-3.01 | **0.001** |
|  |  |  |  | |  | |  |  |
| **Current asthma**  **(N=646)** | 1.43 | 0.85, 2.38 | 0.18 | | 1.76 | | 1.10, 2.83 | **0.02** |
|  |  |  |  | |  | |  |  |
| **Atopy**  **(N=613)** | 1.22 | 0.82-1.79 | 0.33 | | 1.35 | | 0.95-1.94 | 0.098 |
| Boys | BMI-defined | | | PBF-defined | | | | |
|  | **OR** | **[95%CI]** | ***p*-value** | | **OR** | | **[95%CI]** | ***p*-value** |
| **Wheeze**  **(N=339)** | 1.29 | 0.72-2.30 | 0.39 | | 1.41 | | 0.85-2.34 | 0.19 |
|  |  |  |  | |  | |  |  |
| **Current asthma**  **(N=339)** | 1.05 | 0.53-2.08 | 0.88 | | 1.08 | | 0.60-1.95 | 0.80 |
|  |  |  |  | |  | |  |  |
| **Atopy**  **(N=319)** | 1.18 | 0.70-1.98 | 0.54 | | 0.96 | | 0.61-1.51 | 0.86 |
| **Girls** | **BMI-defined** | | | **PBF-defined** | | | | |
|  | **OR** | **[95%CI]** | ***p*-value** | | **OR** | | **[95%CI]** | ***p*-value** |
| **Wheeze**  **(N=307)** | 1.64 | 0.80-3.35 | 0.18 | | 2.81 | | 1.36-5.80 | **0.005** |
|  |  |  |  | |  | |  |  |
| **Current asthma**  **(N=307)** | 2.27 | 1.02-5.07 | **0.05** | | 3.34 | | 1.48-7.56 | **0.004** |
|  |  |  |  | |  | |  |  |
| **Atopy**  **(N=294)** | 1.27 | 0.70-2.29 | 0.44 | | 1.71 | | 0.91-3.22 | 0.10 |
|  |  |  |  | |  |  | |  |
|  |  |  |  |  | |  | |  |

OR: Odds ratio; [95%CI]: Confidence interval 95%; Bold signifies p<0.05

**E**-**Table 2.** *Associations of body adiposity (BMI, PBF and PTF) with pre-bronchodilator lung function - multiple linear regression*

|  | **Overall (N=544)** | | | | **Boys (N=265)** | | | **Girls (N=279)** | | |
| --- | --- | --- | --- | --- | --- | --- | --- | --- | --- | --- |
|  | **β** | **95%CI** | ***p*-value** | | **β** | **95%CI** | ***p*-value** | **β** | **95%CI** | ***p*-value** |
| **BMI**  **z-score**** |  |  |  | |  |  |  |  |  |  |
|  |  |  |  | |  |  |  |  |  |  |
| FEV_1_(L) | 0.030 | 0.008, 0.052 | **0.008** | | 0.019 | -0.014, 0.053 | 0.25 | 0.029 | -0.001, 0.058 | 0.06 |
|  |  |  |  | |  |  |  |  |  |  |
| FVC (L) | 0.066 | 0.037, 0.096 | **<0.001** | | 0.046 | 0.0, 0.093 | 0.05 | 0.062 | 0.029, 0.094 | **<0.001** |
|  |  |  |  | |  |  |  |  |  |  |
|  | **β** | **95%CI** | ***p*-value** *^α^* | | **β** | **95%CI** | ***p*-value** | **β** | **95%CI** | ***p*-value** |
| **BMI*** |  |  |  | |  |  |  |  |  |  |
|  |  |  |  | |  |  |  |  |  |  |
| FEV_1_(L) | 0.007 | 0.000,  0.015 | **0.05** | | 0.005 | -0.006,  0.017 | 0.37 | 0.009 | -0.001, 0.019 | 0.08 |
|  |  |  |  | |  |  |  |  |  |  |
| FVC (L) | 0.018 | 0.009,  0.028 | **<0.001** | | 0.015 | -0.001,  0.032 | 0.06 | 0.021 | 0.010, 0.031 | **<0.001** |
|  |  |  |  | |  |  |  |  |  |  |
| **PBF*** | | | | | | | | | | |
| FEV_1_(L) | -0.002 | -0.006,  0.002 | 0.23 | | -0.004 | -0.009,  0.002 | 0.18 | 0.000 | -0.006,  0.006 | 0.91 |
|  |  |  |  | |  |  |  |  |  |  |
| FVC (L) | 0.001 | -0.004,  0.006 | 0.69 | | -0.002 | -0.010,  0.006 | 0.70 | 0.004 | -0.002,  0.011 | 0.18 |
|  |  |  |  | |  |  |  |  |  |  |
| PTF* | | | | | | | | | | |
| FEV_1_(L) | -0.003 | -0.007,  0.001 | 0.17 | | -0.004 | -0.010,  0.001 | 0.11 | -0.001 | -0.006,  0.005 | 0.76 |
|  |  |  |  | |  |  |  |  |  |  |
| FVC (L) | -0.000 | -0.005, 0.005 | 0.95 | | -0.003 | -0.011,  0.005 | 0.47 | 0.004 | -0.002,  0.010 | 0.19 |
|  |  |  | |  |  |  |  |  |  |  |

#### β: Estimated effect size; 95%CI: 95% Confidence intervals;

*** adjusted for pre/peri-pubertal status and height*

#### *adjusted for pre/peri-pubertal status, exact age, and height

*^α^ also adjusted for gender*

Bold signifies p<0.05

**Legend for E -Figure 1**

**E -Figure 1 *–* Plot for body fat percentage and BMI values for children at age 11 years.**

**Blue triangles – Boys; Red circles – Girls.**
